# Supplementary figures and images for: Long-term prognosis after coronary bifurcation PCI—A nationwide observational study
Source: PLoS One. 2025 Mar 26;20(3):e0317628. doi: 10.1371/journal.pone.0317628 (PMC11940731; doi:10.1371/journal.pone.0317628)

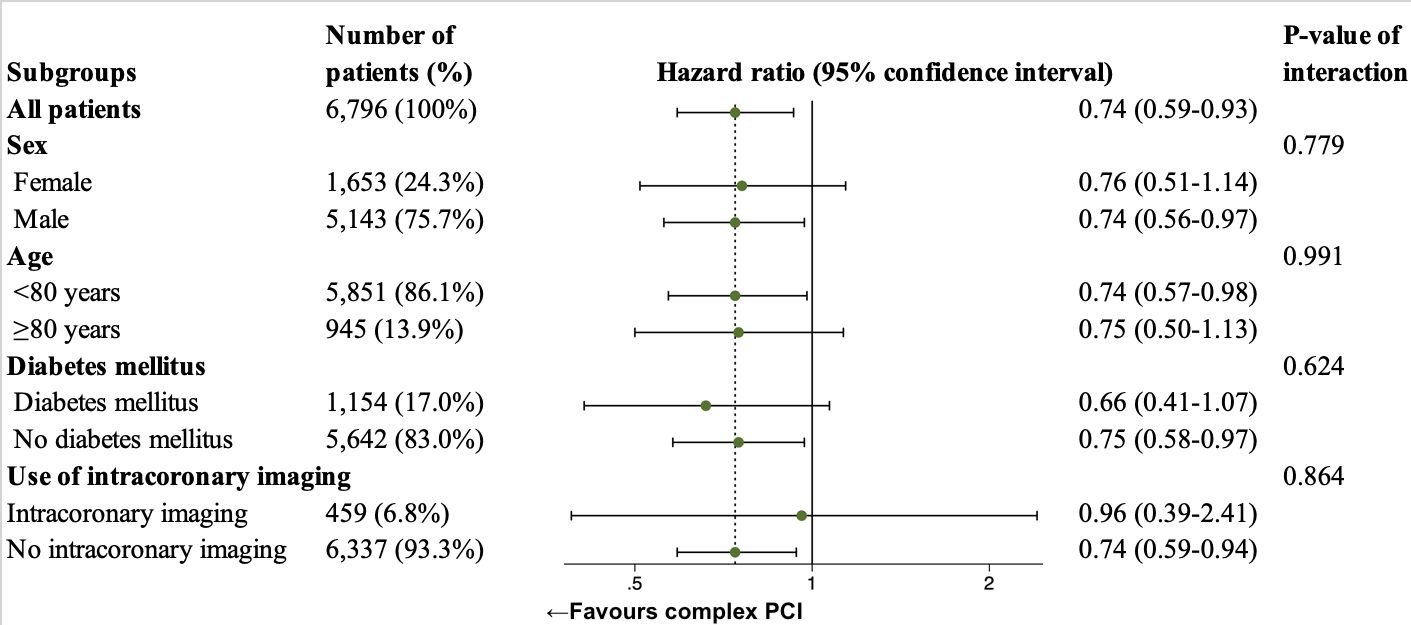

Supplement: S1 Fig — Forest plot illustrating subgroup analysis. Subgroups were analyzed on major adverse clinical events after 1-year follow up. The subgroups were analyzed using a multivariable Cox regression model including age, sex, inclusion year, diabetes mellitus, hypertension, hyperlipidemia, smoking status (non-smokers, previous smokers, and active smokers), use of intracoronary image, indication (stable coronary artery disease, unstable angina, NSTEMI and STEMI) and lesion classification (B1 bifurcation, B2 bifurcation and C bifurcation). NSTEMI = non-ST-elevation myocardial infarction; PCI = percutaneous coronary intervention; STEMI = ST-elevation myocardial infarction. (TIF) [file pone.0317628.s001.tif]

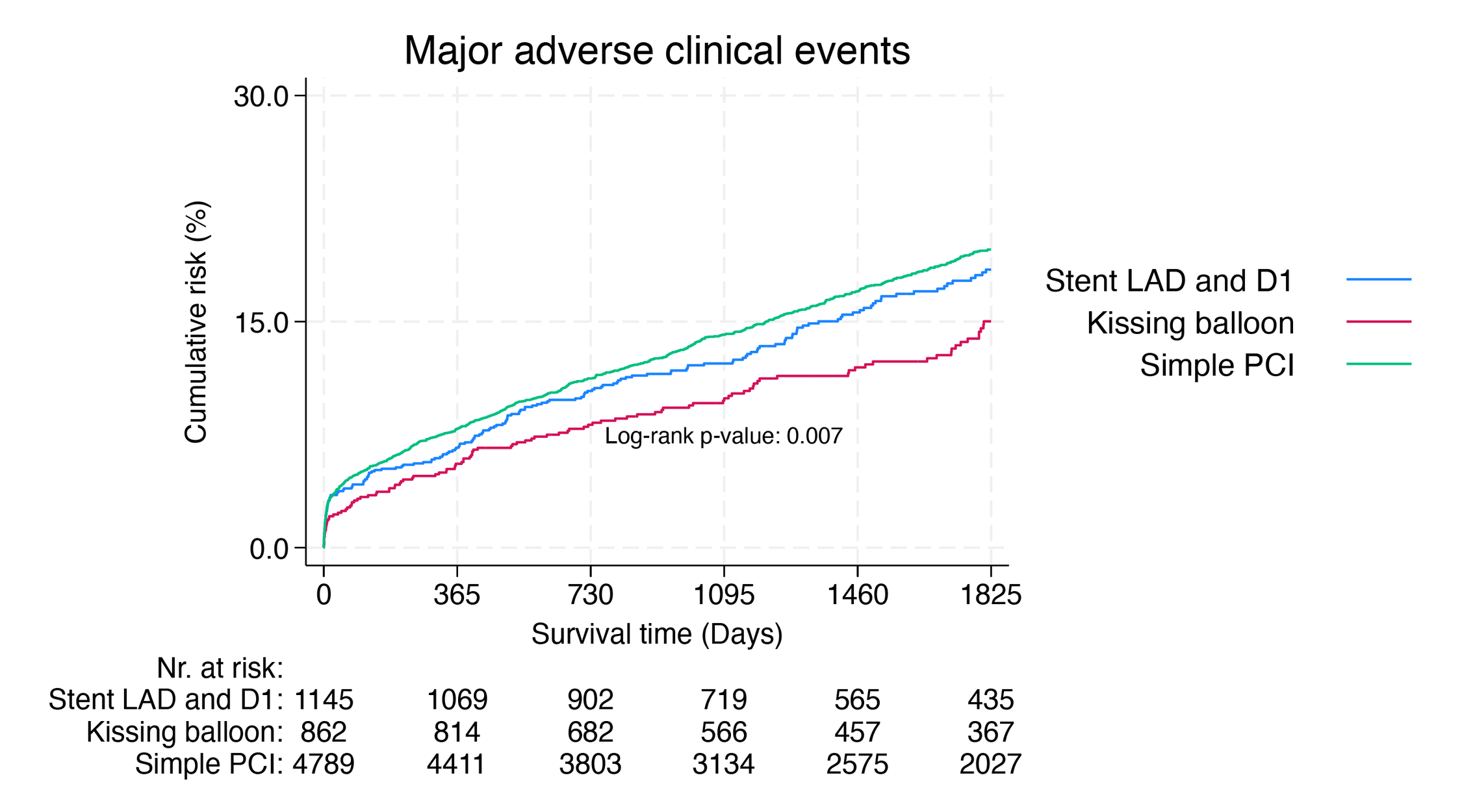

Supplement: S2 Fig — Kaplan-Meier curves illustrating the 5-year event rate of major adverse clinical events comparing Simple PCI, kissing balloon technique and a two-stent approach. Major adverse cardiovascular event was defined as all-cause mortality or myocardial infarction. D1 = first diagonal branch; LAD = left anterior descending artery; PCI = percutaneous coronary intervention. (TIF) [file pone.0317628.s002.tif]
