# Supplementary material for: Long-term prognosis after coronary bifurcation PCI—A nationwide observational study
Source: PLoS One. 2025 Mar 26;20(3):e0317628. doi: 10.1371/journal.pone.0317628 (PMC11940731; doi:10.1371/journal.pone.0317628)
Supplement: S1 Table — CABG = coronary artery by-pass graft surgery; ICD = international classification of diseases; PCI = percutaneous coronary intervention; SCAAR = Swedish Coronary Angiography and Angioplasty registry. (DOCX) [file pone.0317628.s003.docx]

**Supplementary Table 1. Outcome definition**

| **Outcome** | **Source** | **Definition** |
| --- | --- | --- |
| Major adverse clinical events | National population registry and SCAAR | All-cause mortality or myocardial infarction |
| All-cause mortality | National population registry | All-cause death |
| Myocardial infarction | SCAAR and national patient registry | First 30 days, angiographically verified myocardial infarction. Post 30 days, new registration with a discharge diagnosis of myocardial infarction according to the fourth universal definition of myocardial infarction, ICD-10: I21–I22. |
| Target segment revascularization or CABG | SCAAR | Any repeat revascularization with PCI in segment 6, segment 9 or segment 7 or an event of CABG. |
| Definite stent thrombosis | SCAAR | Any angiographically verified stent thrombosis |
| Bleeding event | National patient registry | Haemorrhagic stroke:  ICD-10: I60-I62.  Gastrointestinal bleeding:  ICD-10: K226, K250, K252, K254, K256, K260, K262, K264, K266, K270, K272, K274, K276, K280, K282, K284, K286, K290, K625, K920, K921, K922, I850.  Anaemia-related bleeding:  ICD-10: D629, D500.  Other bleeding:  ICD-10: N421, N938, N939, N950, R041, R042, R048, R049, R210, R319, R210, T810, N501A |
| Stroke | National patient registry | ICD-10: I63.0–I63.6. |

CABG = coronary artery by-pass graft surgery; ICD = international classification of diseases; PCI = percutaneous coronary intervention; SCAAR = Swedish Coronary Angiography and Angioplasty registry.
