# Supplementary material for: Long-term prognosis after coronary bifurcation PCI—A nationwide observational study
Source: PLoS One. 2025 Mar 26;20(3):e0317628. doi: 10.1371/journal.pone.0317628 (PMC11940731; doi:10.1371/journal.pone.0317628)
Supplement: S2 Table — E-values were calculated for all outcomes with statistically significant differences between Simple PCI and Complex PCI. E-values were calculated using the adjusted and unadjusted effect estimates from Fig 2. Outcome. CI = confidence interval; MACE = major adverse clinical events. (DOCX) [file pone.0317628.s004.docx]

**Supplementary Table 2. E-values**

|  | **E-value for point estimate** | **E-value for upper bound of CI** |
| --- | --- | --- |
| **1-year** |  |  |
| MACE | Unadjusted: 1.90  Adjusted: 2.03 | Unadjusted: 1.29  Adjusted: 1.36 |
| All-cause mortality | Unadjusted: 2.19  Adjusted: 2.10 | Unadjusted: 1.40  Adjusted: 1.17 |
| **5-year** |  |  |
| MACE | Unadjusted: 1.53  Adjusted: 1.54 | Unadjusted: 1.23  Adjusted: 1.21 |
| All-cause mortality | Unadjusted: 1.95  Adjusted: 1.90 | Unadjusted: 1.45  Adjusted: 1.35 |

E-values was calculated for all outcomes with statistically significant differences between Simple PCI and Complex PCI. E-values was calculated using the adjusted and unadjusted effect estimates from *Figure 2. Outcome.* CI = confidence interval; MACE = major adverse clinical events.
