# Supplementary material for: Long-term prognosis after coronary bifurcation PCI—A nationwide observational study
Source: PLoS One. 2025 Mar 26;20(3):e0317628. doi: 10.1371/journal.pone.0317628 (PMC11940731; doi:10.1371/journal.pone.0317628)
Supplement: Attachment — Submitted filename: Rebuttal PlosOne.docx [file pone.0317628.s006.docx]

**Editorial Board Comments for the Author.**

We thank the editor for their valuable suggestions and helpful comments. The manuscript has been formatted in accordance with the points outlined below. We took the liberty of formatting it before activating track changes and then enabled track changes for subsequent modifications to the manuscript text.

**1. Please ensure that your manuscript meets PLOS ONE's style requirements, including those for file naming. The PLOS ONE style templates can be found at**

[**https://journals.plos.org/plosone/s/file?id=wjVg/PLOSOne_formatting_sample_main_body.pdf**](https://journals.plos.org/plosone/s/file?id=wjVg/PLOSOne_formatting_sample_main_body.pdf) **and**

[**https://journals.plos.org/plosone/s/file?id=ba62/PLOSOne_formatting_sample_title_authors_affiliations.pdf**](https://journals.plos.org/plosone/s/file?id=ba62/PLOSOne_formatting_sample_title_authors_affiliations.pdf)

Reply: we have formatted the manuscript in accordance with PLOS ONE's style requirements.

**2. We note that you have indicated that there are restrictions to data sharing for this study. For studies involving human research participant data or other sensitive data, we encourage authors to share de-identified or anonymized data. However, when data cannot be publicly shared for ethical reasons, we allow authors to make their data sets available upon request. For information on unacceptable data access restrictions, please see** [**http://journals.plos.org/plosone/s/data-availability#loc-unacceptable-data-access-restrictions**](http://journals.plos.org/plosone/s/data-availability#loc-unacceptable-data-access-restrictions)**.**

**Before we proceed with your manuscript, please address the following prompts:**

**a) If there are ethical or legal restrictions on sharing a de-identified data set, please explain them in detail (e.g., data contain potentially identifying or sensitive patient information, data are owned by a third-party organization, etc.) and who has imposed them (e.g., a Research Ethics Committee or Institutional Review Board, etc.). Please also provide contact information for a data access committee, ethics committee, or other institutional body to which data requests may be sent.**

**b) If there are no restrictions, please upload the minimal anonymized data set necessary to replicate your study findings to a stable, public repository and provide us with the relevant URLs, DOIs, or accession numbers. Please see** [**http://www.bmj.com/content/340/bmj.c181.long**](http://www.bmj.com/content/340/bmj.c181.long) **for guidelines on how to de-identify and prepare clinical data for publication. For a list of recommended repositories, please see** [**https://journals.plos.org/plosone/s/recommended-repositories**](https://journals.plos.org/plosone/s/recommended-repositories)**. You also have the option of uploading the data as Supporting Information files, but we would recommend depositing data directly to a data repository if possible.**

**Please update your Data Availability statement in the submission form accordingly.**

Reply. Unfortunately, Swedish law and legal requirements do not permit immediate data sharing. In line with previous publications in Plos One from our registry (e.g. PMID: 35025950), we have included a statement addressing this issue (P 14):

*Data Availability*

*SWEDEHEART does not allow individual data sharing to third parties. Access to aggregated data might be granted following review by the SWEDEHEART steering committee. Such requests can be submitted to the SWEDEHEART steering committee for consideration. For contact details see* [*https://www.ucr.uu.se/swedeheart/kontakt/styrgrupp*](https://www.ucr.uu.se/swedeheart/kontakt/styrgrupp)*.*

**3. PLOS requires an ORCID iD for the corresponding author in Editorial Manager on papers submitted after December 6th, 2016. Please ensure that you have an ORCID iD and that it is validated in Editorial Manager. To do this, go to ‘Update my Information’ (in the upper left-hand corner of the main menu), and click on the Fetch/Validate link next to the ORCID field. This will take you to the ORCID site and allow you to create a new iD or authenticate a pre-existing iD in Editorial Manager.**

Reply. The corresponding author (Ole Frobert) has updated this information.

**4. Please include a separate caption for each figure in your manuscript.**

Reply. We have done this in the revised version.

**5. Please include your tables as part of your main manuscript and remove the individual files. Please note that supplementary tables should remain as separate "supporting information" files.**

Reply. We have done this in the revised version.

**6. We notice that your supplementary figures are uploaded with the file type 'Figure'. Please amend the file type to 'Supporting Information'. Please ensure that each Supporting Information file has a legend listed in the manuscript after the references list.**

Reply. We have changed this in the revised version and amended file types as requested.

**7. Please review your reference list to ensure that it is complete and correct. If you have cited papers that have been retracted, please include the rationale for doing so in the manuscript text, or remove these references and replace them with relevant current references. Any changes to the reference list should be mentioned in the rebuttal letter that accompanies your revised manuscript. If you need to cite a retracted article, indicate the article’s retracted status in the References list and also include a citation and full reference for the retraction notice.**

Reply. We have reviewed the reference list and not made any changes to the reference list in the revised version.

**Review Comments to the Author**

**Reviewer #1: This real world data from a comprehensive registry is very valuable. Although at variance with what randomized clinical trials suggest it is reassuring that when a patient is deemed to need 2 stents or kissing balloon dilation for the LAD/D1 bifurcation, the long term result is good and should not prevent interventional cardiologists from performing this procedure when deemed required to achieve an acceptable result.**

We thank the reviewer for this kind feedback.

**Reviewer #2: I would like to congratulate you on conducting such a comprehensive and valuable study that contributes significantly to the field of coronary bifurcation PCI. The use of the SWEDEHEART registry and the long-term follow-up data strengthens the robustness of your findings. However, I would like to kindly point out a few considerations. While you acknowledge the potential impact of selection bias and residual confounding, the absence of Medina bifurcation lesion classification in the dataset may have limited the granularity of your analysis, potentially affecting the comparison between the treatment strategies. Although you briefly discuss this limitation, further consideration of their potential impact could add depth to the interpretation of your findings. I believe your study provides valuable insights and the results remain compelling. I look forward to seeing future studies that may address these factors to further enhance the clinical relevance of the findings.**

We thank the reviewer for valuable suggestions and helpful comments. In the Discussion under limitations we have added (P13, L305):

*As lesion morphology assessed by the Medina classification could influence the selection and outcomes of PCI strategies, its absence may affect the interpretation of the comparative effectiveness of simple versus complex PCI approaches.*

**Reviewer #3: The manuscript provides valuable insights into the long-term outcomes of complex versus simple PCI in the treatment of LAD-D1 bifurcation lesions, grounded in real-world data. Despite its limitations, such as the observational nature and incomplete lesion classification, the study’s findings are significant for clinical practice, suggesting that complex PCI may provide better outcomes in routine care. The authors made it clear that the use of intravascular imaging was deficient, which should not be the case nowadays.**

We thank the reviewer for this kind feedback.

**Reviewer #4: The study is exhaustive with large number of patients, and the article is well written with good statistics.**

We thank the reviewer for valuable suggestions and helpful comments.

**1. Since the study was predominantly performed in LAD D1 bifurcation, the heading can be modified as LAD D1 bifurcation lesions.**

Reply. We are not completely opposed to changing the title, and if the editors favor a change, we will comply. However, we prefer the current title because it highlights LAD/D1 bifurcations as a case in point to illustrate the potential maximal impact of different stenting techniques. We aimed to make this clear in the Introduction (P4, L110-13):

We chose to focus on the LAD-D1 bifurcation because it is well-defined anatomically and due to its critical importance in supplying a substantial portion of the myocardium, with the LAD perfusing the anterior wall, septum, and apex, and the D1 perfusing the lateral aspect of the left ventricle.

**2. The stent characteristics is not available in both categories, especially if they are all drug eluting or any bare metal stents were used or not, in both categories. If the information is not available it can be mentioned as a minor limitation in study.**

Thanks. Only drug-eluting stents were used. This is mentioned in the abstract (the word ‘stent’ was missing, now corrected) and methods (P5, L139).

**3. In simple stenting, the patients were slightly older, higher incidence of diabetes, lesser intake of statins, increased female patients, more early recruitment (<2015) and comparatively double the number of patients which could have influenced the results. This can be mentioned a minor limitation in discussion.**

Reply. Thanks. In the revised version we have mentioned these differences (except diabetes, which did not differ between groups) under limitations (P13, L310-15):

*For example, patients in the simple PCI group were slightly older, included more women, and were less frequently prescribed statins. Additionally, this group had a larger sample size, with more patients recruited before 2015, which may reflect temporal changes in practice and outcomes. These differences could have influenced the observed results and should be considered when interpreting the findings.*
